# Coordination of cohabiting phage elements supports bacteria–phage cooperation

**DOI:** 10.1038/s41467-019-13296-x

**Published:** 2019-11-21

**Authors:** Tal Argov, Shai Ran Sapir, Anna Pasechnek, Gil Azulay, Olga Stadnyuk, Lev Rabinovich, Nadejda Sigal, Ilya Borovok, Anat A. Herskovits

**Affiliations:** The School of Molecular Cell Biology and Biotechnology, Faculty of Life Sciences, Tel Aviv University, Ramat Aviv, Tel Aviv 69978 Israel

**Keywords:** Bacterial genetics, Bacteriophages, Pathogens, Phage biology

## Abstract

Bacterial pathogens often carry multiple prophages and other phage-derived elements within their genome, some of which can produce viral particles in response to stress. *Listeria monocytogenes* 10403S harbors two phage elements in its chromosome, both of which can trigger bacterial lysis under stress: an active prophage (ϕ10403S) that promotes the virulence of its host and can produce infective virions, and a locus encoding phage tail-like bacteriocins. Here, we show that the two phage elements are co-regulated, with the bacteriocin locus controlling the induction of the prophage and thus its activity as a virulence-associated molecular switch. More specifically, a metalloprotease encoded in the bacteriocin locus is upregulated in response to stress and acts as an anti-repressor for CI-like repressors encoded in each phage element. Our results provide molecular insight into the phenomenon of polylysogeny and its intricate adaptation to complex environments.

## Abstract (web summary)

Bacterial pathogens often carry multiple phage-derived elements within their genome. Here, the authors show that two phage elements are co-regulated in *Listeria monocytogenes*, the first one controlling the induction of the second one, which in turn regulates virulence of their bacterial host.

## Introduction

Most bacterial pathogens are lysogens, carry prophages within their chromosome, in many cases more than one^1^. In times of stress (e.g., SOS or starvation), these prophages can turn into lytic production (i.e., produce infective virions) and kill their host bacterium in a matter of minutes^2,3^, and yet it is unclear how pathogens manage to survive this internal threat given the stresses engendered during their invasion of mammalian cells.

*Listeria monocytogenes* (*Lm*) is a saprophyte that is highly abundant in the environment and a serious human pathogen that can cause listeriosis disease^4,5^. It is a facultative intracellular pathogen that invades a wide variety of mammalian cells^6^. Upon invasion, *Lm* resides within a vacuole (or a phagosome in phagocytic cells), from which it escapes into the host cell cytosol in order to replicate^5,7^. In the cytosol, the bacteria exploit the host actin polymerization machinery to propel themselves on actin filaments in order to spread from cell to cell^8^. It has long been known that certain *Lm* strains, especially those associated with outbreaks of foodborne illness, carry a ~40-kb-long infective prophage of the *Siphoviridae* family, integrated within the *comK* gene^9^. The prototype of these *comK* phages, phage A118 of WSLC1118 *Listeria* strain, was the first to be sequenced^9^, but there are now over 850 A118-like phages that have been sequenced, together with their host chromosome. Because of the phage insertion, the listerial *comK* gene was considered to be non-functional. In *Bacillus subtillis*, ComK functions as the master transcriptional activator of the competence system (the *com* genes), a system that is known to facilitate DNA uptake^10,11^. In previous studies we noticed that the *com* genes of *Lm* 10403S are highly transcribed during intercellular growth in macrophage cells. Further investigation revealed that some of the *com* genes *i.e., comEC* and *comG* (encoding the Com system membrane channel and pseudo-pilus, respectively), play a role in the escape of *Lm* from the phagosomes and hence promote *Lm* intracellular growth^12^. The expression of the *com* genes during *Lm* infection of macrophages was found to require an intact *comK* gene, formed by precise excision of the ϕ10403S-prophage. The prophage excision was strongly induced when the bacteria were located within the macrophage phagosomes, but in contrast to classic phage induction, this did not lead to the production of progeny virions and bacterial lysis^12^. These observations demonstrated an intriguing adaptive (cooperative) behavior of the prophage to the intracellular life style of its host, where it apparently could serve as a molecular switch that regulates bacterial gene expression to promote virulence. We termed this type of phage behaviour *active lysogeny*, to describe cases where prophages function as active regulatory DNA elements without triggering their lytic cycle^13,14^.

While the forces that drive such unusual phage adaptive behaviors are not always clear, the evolution of bacteria–phage cooperative interactions is even more intriguing, since in nature bacteria rarely interact with a single phage. With the massive accumulation of genomic data, it is now evident that most bacterial genomes carry multiple prophages (infective and defective), a phenomenon called polylysogeny^1,15–18^. Notably, polylysogeny is widespread in bacterial pathogens^15,17,19–21^, raising the question of how the pool of prophages that inhabit the genome manage to synchronize their behaviour and conform to the pathogenic life style of their host (i.e., avoid killing their host within the mammalian niche). In light of this question, we asked whether the ϕ10403S cooperative behavior observed within mammalian cells could be coordinated with the other phage elements that inhabit the chromosome, particularly those able to trigger bacterial lysis.

Here we show that the *Lm* strain 10403S carries two lytic phage elements, one producing infective virions (ϕ10403S) and the other cryptic producing bacteriocins, that are fully synchronized under SOS conditions and during *Lm* infection of mammalian cells. The two phage elements are tightly co-regulated, exhibiting a regulatory hierarchy in which the cryptic element controls ϕ10403S-prophage, rendering it non-autonomous. Our findings reveal an inter-phage cross-regulatory interaction that coordinates co-habiting lytic phage elements, and promotes their cooperation with the host in complex environments, such as within the mammalian niche.

## Results

### *Lm* 10403S harbors two fully synchronized lytic phage elements

**Fig. 1:**
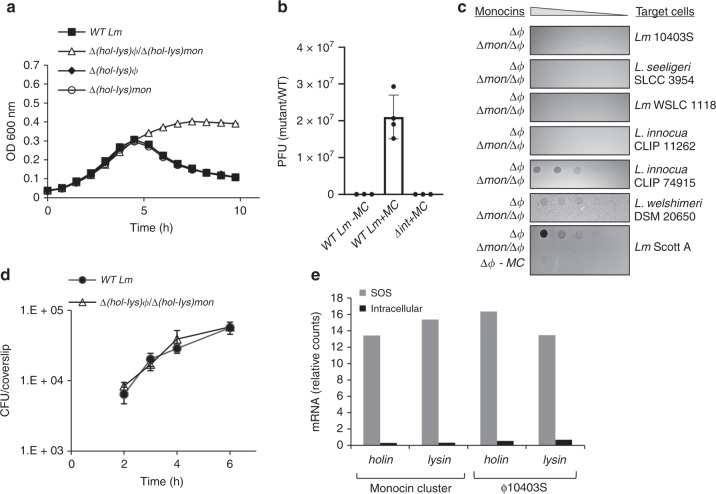
The *Lm* 10403S strain harbors two functional lytic phage elements. **a** Growth analysis of WT *Lm* and mutants harboring deletions of the elements lysis modules; *LMRG_01552–4* of ϕ10403S (*Δ(hol-lys)*_ϕ_), and *LMRG_02377-8* of monocin cluster (*Δ(hol-lys)*_*mon*_) or a mutant deleted of both lysis modules (*Δhol-lys)*_ϕ_*/Δ(hol-lys)*_*mon*_), in the presence of mitomycin C (MC). Growth analysis of the mutants without MC is presented in Supplementary Fig. 1. Error bars represent the standard deviation of three independent biological repeats, and are hidden by the symbols. **b** Plaque forming assay of WT *Lm* and a mutant deleted for the ϕ10403S integrase gene, *Δint* (*LMRG_01511*) with MC treatment (+MC). Virions obtained from MC treated bacterial cultures (4 h post MC treatment) or from bacteria grown to exponential phase (3 h, OD_600_ 0.5) without MC treatment (*WT Lm* -MC), tested on an indicator strain for plaque forming units (PFU). Error bars represent the standard deviation of three independent experiments. **c** A monocin killing assay performed on monocins obtained from MC treated bacterial cultures of the *Lm* ϕ10403S-phage cured strain (Δϕ) and a mutant lacking both the monocin cluster (*LMRG_02362-02378*) and ϕ10403S-phage (Δ*mon/*Δϕ), as a control. Five-fold serial dilutions of filtered supernatants (containing monocins) were applied on a lawn of different *Listeria* strains (target cells), and incubated for 1–2 days. The dark zones of growth inhibition indicate monocin killing activity. The experiment was performed three times. Monocins from Δϕ bacteria that were not treated with MC (Δϕ –MC) are shown at the bottom, as a reference. **d** Intracellular growth analysis of WT *Lm* and a deletion mutant lacking both lysis modules (*Δ(hol-lys)*_ϕ_*/Δ(hol-lys)*_*mon*_) in bone marrow derived macrophage (BMDM) cells. Growth curves represent one biological replicate, more independent experiments are shown in the source data file. Error bars represent standard deviation of a technical triplicate, sometimes hidden by the symbols. **e** Transcription analysis of the two phage elements holin and endolysin genes under SOS and intracellular growth conditions using NanoString technology (6 h post BMDM infection). Transcription levels are presented as fold change of relative counts for the indicated gene mRNA, relative to the levels observed during lysogeny (i.e., exponentially grown bacteria in BHI medium). Data represent 3 independent experiments. Source data are provided as a Source Data file.

We searched the *Lm* 10403S chromosome for the presence of additional phage elements that have the potential to trigger bacterial lysis. We searched for phage-like *holin* and *endolysin* genes (*hol-lys* in short) and identified two pairs, one located within ϕ10403S-prophge and the other within a phage-related element located in a separate chromosomal region. This element was previously shown in other *Lm* strains to produce F-Type bacteriocins (monocins), which are phage tail-like structures that are capable of killing closely related listerial strains^22,23^. These monocins were shown to be produced under SOS conditions and to be liberated by the bacterial lysis that is driven by the element encoded holin and endolysin^23,24^. As a first step, we examined the capacity of each *hol-lys* module to trigger bacterial lysis in response to treatment with mitomycin C (MC), a DNA damaging agent known to trigger the SOS response. Mutants where each *hol-lys* module was deleted (i.e., ∆(*hol-lys*)_ϕ_ and ∆(*hol-lys*)_*mon*_ of ϕ10403S-prophage and the monocin element, respectively), as well as a double mutant with both modules deleted, (∆(*hol-lys*)_ϕ_/∆(*hol-lys*)_*mon*_), were generated and monitored for growth in the presence of MC compared to wild type (WT) bacteria. As shown in Fig. 1a (and Supplementary Fig. 1), we found the two *hol-lys* modules to be equally potent and redundant in triggering bacterial lysis under SOS conditions. Furthermore, these modules proved to be the only determinates that lyse the bacteria, as the double *hol-lys* mutant did not cause bacterial lysis in the presence of MC (Fig. 1a). In order to examine the capacity of the two phage elements to produce lytic particles, i.e., ϕ10403S infective virions and monocins, the activity of each particle was evaluated using a plaque assay and a bacteriocin-killing assay, respectively. The results of these assays demonstrated that both elements are functional, concomitantly producing and releasing infective virions and monocins under SOS conditions (Fig. 1b, c). A mutant deleted of ϕ10403S integrase gene (∆*int*) was used as a control for ϕ10403S production of infective virions (Fig. 1b). Notably, the *Lm* 10403S monocins could kill a variety of *Listeria* strains and species, e.g., *Lm* Scott A, *L. innocua* CLIP 74915, and *L. welshimeri* DSM 20650 (but not the parental strain), an activity that was independent of ϕ10403S-prophage (Fig. 1c). Having established that *Lm* 10403S carries two functional lytic phage elements that are synchronized during SOS, we next examined their behaviour during *Lm* infection of mammalian cells. We first evaluated whether the two phage element trigger bacterial lysis during *Lm* infection of macrophage cells. As shown in Fig. 1d, the ∆(*hol-lys*)_ϕ_/∆(*hol-lys*)_*mon*_ double mutant grew as well as WT bacteria in bone marrow derived macrophages (BMDMs), suggesting that both *hol-lys* modules are effectively repressed within the intracellular niche. Transcriptional analysis of the two *hol-lys* gene pairs under SOS and intracellular growth conditions corroborated this premise, and demonstrated that the two phage derived lysis modules are fully coordinated extracellularly and intracellularly, i.e., activated upon SOS and repressed upon infection of mammalian cells (Fig. 1e).

### The monocin element mediates ϕ10403S excision in mammalian cells

**Fig. 2:**
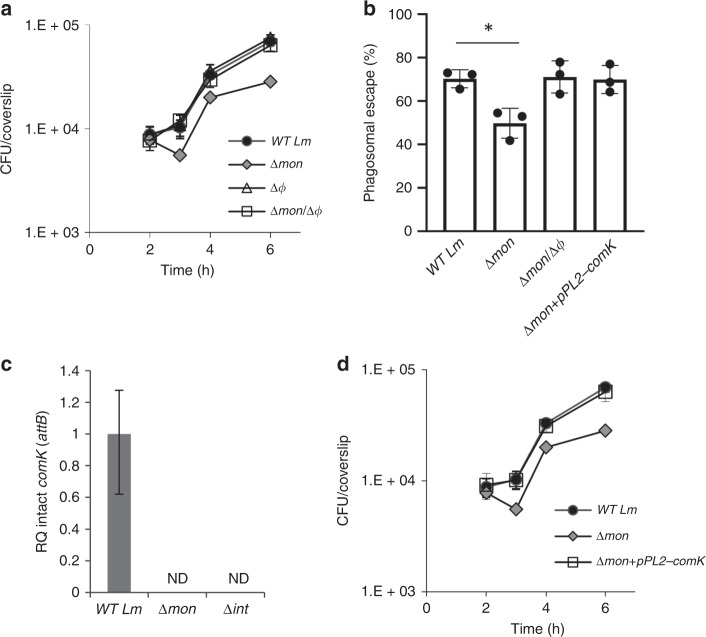
The monocin element affects ϕ10403S excision within macrophage cells. **a** Intracellular growth analysis of WT *Lm* and mutants lacking each of the phage elements, Δ*mon* and Δ*ϕ* (monocin cluster and ϕ10403S, respectively), and a double mutant lacking both phage elements Δ*mon/*Δ*ϕ*, in BMDM cells. Growth curves represent one biological replicate, more independent experiments are shown in the source file. Error bars represent standard deviation of triplicate samples, and are hidden by the symbols. **b** A bacterial phagosomal escape assay. Percentage of bacteria that escaped the macrophage phagosomes at 2.5 h post infection, as determined by a microscope fluorescence assay. Macrophages were infected with WT *Lm*, Δ*mon* and Δ*mon/*Δϕ bacteria, as well as with a Δ*mon* mutant that was complemented with an intact *comK* gene on the pPL2 plasmid (Δ*mon+*pPL2*-comK*). The data is a mean of three independent experiments. The error bar represent standard deviation. Asterisk (*) indicates statistical significance of *p* = 0.01 calculated using Student's *t*-test. **c** qRT-PCR analysis of intact *comK* gene (representing ϕ10403S *attB* site) in WT *Lm* and indicated mutants grown intracellularly in BMDM cells (6 h post infection). Presented as relative quantity (RQ), relative to the levels in WT bacteria. The data represent three independent experiments. Error bars indicate a 95% confidence interval. **d** Intracellular growth analysis of WT *Lm*, *Δmon* and the *Δmon* mutant complemented with an intact *comK* gene with its native promoter (Δ*mon+*pPL2*-comK*) in BMDM cells. Growth curves represent one biological replicate, more independent experiments are shown in Supplementary Fig. 2 and in the source data file. Error bars represent standard deviation of triplicate samples, and are hidden by the symbols. Source data are provided as a Source Data file.

Since we had previously described that genomic excision of ϕ10403S serves as a molecular switch of *comK* gene expression during *Lm* infection of macrophage cells^12^, we were also interested in examining the effect of the monocin element on *Lm* intracellular growth. For this purpose, we generated a ∆*mon* mutant that is deleted of all 17 genes of the monocin cluster (see below), and monitored the intracellular growth of this mutant within macrophage cells. Surprisingly, we found the ∆*mon* mutant to exhibit defects in both intracellular growth and phagosomal escape when compared to WT bacteria (Fig. 2a, b). Notably, these phenotypes were completely dependent on the presence of ϕ10403S within the chromosome, since a mutant lacking both phage elements (∆*mon*/∆*ϕ*, harboring an intact *comK* gene) behaved like WT bacteria (Fig. 2a, b). Of note, we previously showed that ϕ10403S itself is not required for *Lm* intracellular growth^12^ (Fig. 2a). A strain lacking ϕ10403S (∆*ϕ*) grew as well as WT bacteria in macrophage cells, because it carries a functional *comK* gene. These observations raised the possibility that the monocin element affects ϕ10403S genomic excision, and thereby influences the expression of *comK*, which promotes *Lm* phagosomal escape and intracellular growth^12^. To test the hypothesis that the monocin element affects ϕ10403S-excision, we examined ϕ10403S-excision in ∆*mon* bacteria growing intracellularly in macrophage cells, by using quantitative real-time PCR (qRT-PCR) to evaluate the formation of an intact *comK* gene. This assay also quantitates the re-formation of the *attB* recognition site, which is located within the intact *comK* gene. The data demonstrated that ϕ10403S cannot excise its genome in the absence of the monocin element, as not even a single copy of an intact *comK* gene was detected in *∆mon* bacteria. This phenotype was reminiscent of the ∆*int* mutant, which lacks the ϕ10403S integrase gene, which is essential for prophage excision (Fig. 2c). To confirm that the intracellular growth defect of ∆*mon* bacteria is due to their inability to form a functional (intact) *comK* gene, we used the integrative pPL2 plasmid to reintroduce an intact copy of *comK* gene under its native promoter (pPL2-*comK*). As demonstrated in Fig. 2b, d, the intact copy of *comK* fully complemented the ∆*mon* intracellular defects (i.e., phagosomal escape and intracellular growth), supporting the premise that the monocin element plays a critical role in ϕ10403S genomic excision, and hence in *comK* gene expression, during *Lm* infection of mammalian cells (additional supporting data in Supplementary Fig. 2).

Of note, we suggested before that ϕ10403S-excision in mammalian cells is a transient event that is followed by the phage re-integration to *comK*. To evaluate this re-integration and the occurrence of phage-cured bacteria in macrophage cells, we cloned the pheS* counter selection gene^24^ (encoding a mutated phenylalanyl-tRNA synthetase) into ϕ10403S genome and counter-selected for phage-cured bacteria upon *Lm* intracellular growth (see material and methods for more details). Remarkably, we found that ϕ10403S re-integrates into *comK* in a highly efficient manner, as the rate of phage-loss was 1 to 500,000 bacteria. Notably, under SOS and stationary growth phage-cured bacteria were not detected (phage loss rate is <1–10^–^^9^).

### The monocin encoded metalloprotease controls ϕ10403S excision

**Fig. 3:**
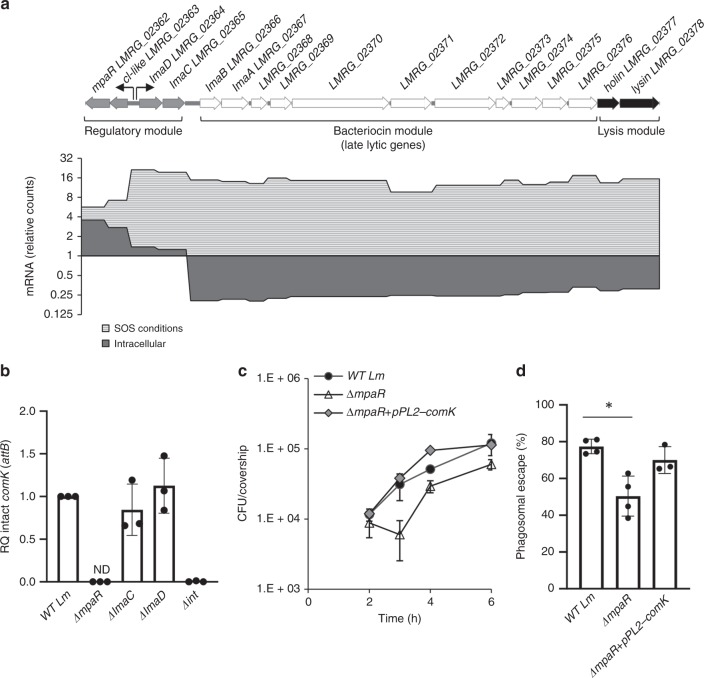
MpaR is required for ϕ10403S excision. **a** Upper panel: schematic representation of the monocin locus. The *mon* consists of 17 genes comprising: a regulatory module, a bacteriocin module encoding tail-like structures, and a lysis module. Lower panel: transcription analysis of the monocin cluster genes under SOS and intracellular growth (in BMDM cells) conditions, using NanoString technology. Transcription levels are presented as relative counts, relative to the levels observed at the lysogenic state (i.e., exponentially grown bacteria in BHI medium at 37˚C). Data represent three independent experiments. **b** qRT-PCR analysis of intact *comK* gene (representing ϕ10403S *attB* site) in WT *Lm* and indicated mutants grown intracellularly in BMDM cells (6 h post infection). Presented as relative quantity (RQ), relative to the levels in WT bacteria. The data is a mean of three independent experiments. Error bars indicate a standard deviation. **c** Intracellular growth analysis of WT *Lm*, Δ*mpaR* and Δ*mpaR* mutant complemented with an intact *comK* gene under its native promoter (Δ*mpaR+*pPL2-*comK*). Growth curves represent one biological replicate, more independent experiments are shown in Supplementary Fig. 2 and in the source file. Error bars represent standard deviation of triplicate samples, sometimes are hidden by the symbols. **d** A bacterial phagosomal escape assay. Percentage of bacteria that escaped the macrophage phagosomes at 2.5 h post infection, as determined by a microscope fluorescence assay. Macrophages were infected with WT *Lm*, Δ*mpaR* and Δ*mpaR* mutant complemented with an intact *comK* gene on the pPL2 plasmid (Δ*mpaR+*pPL2*-comK*). The data is a mean of three independent experiments. The error bar represent standard deviation. Asterisk (*) indicates statistical significance of *p* < 0.01 calculated using Student's *t*-test. Source data are provided as a Source Data file.

Having discovered an intriguing link between the two phage elements, we next sought to identify the exact determinants within the monocin gene cluster that are responsible for ϕ10403S genomic excision. The 17 genes of the monocin element are organized in three classic modules, possibly a remnant of an ancient ancestral prophage (Fig. 3a). The predicted “regulatory module” encodes four putative regulatory factors, the “late-lytic gene module” that is the “bacteriocin module”, encodes a phage tail-like, tape measure and receptor-binding proteins (missing capsid genes), and the “lysis module” encodes holin and endolysin. Importantly, the monocin element lacks a classic “early-lytic gene module”, which typically includes genes that mediate phage DNA excision and replication, and therefore is unable to excise its genome under SOS conditions (Supplementary Fig. 3). To decipher which genes of the monocin cluster play a role in *Lm* infection of macrophage cells, we analyzed the transcription profile of the monocin element in intracellularly grown bacteria compared to bacteria grown under SOS conditions in the rich medium, brain heart infusion (BHI). As expected, under SOS conditions the entire monocin element was upregulated, demonstrating enhanced transcription of the regulatory, bacteriocin and lysis modules (Fig. 3a). In contrast, in macrophage cells, only the regulatory module was upregulated while the bacteriocin and the lysis modules were specifically repressed, suggesting that the monocin regulatory genes may affect ϕ10403S excision within the macrophage cells. We noticed that the monocin regulatory module possesses a typical organization of a lysogenic-lytic molecular switch^25^, harboring two oppositely directed promoters transcribing on one direction, a putative CI-like repressor and a metalloprotease of DUF955 family^26^, named here *cI-like* and *mpaR* respectively, and on the other direction two putative “early” genes, *lmaD* and *lmaC*, of unknown function (Fig. 3a). To examine which product of these genes affects ϕ10403S-excision in macrophage cells, the formation of intact *comK* gene was measured in a set of mutants where each one of the genes was deleted in turn (i.e.*, ∆mpaR*, *∆lmaD* and ∆*lmaC*), except for the *cI-like* repressor gene. Deletion of the *cI-like* repressor results in the lethal activation of the monocin and lysis genes. The data clearly identified MpaR, the monocin-encoded putative metalloprotease, as the factor that mediates ϕ10403S excision in macrophage cells (Fig. 3b). In line with this finding, a *∆mpaR* mutant exhibited an intracellular growth defect and a phagosomal escape defect in macrophage cells, which were complemented by introducing, in trans, an intact copy of the *comK* gene (pPL2-*comK*, Fig. 3c, d and Supplementary Fig. 2). Taken together these results demonstrate that MpaR plays an essential role in mediating ϕ10403S excision during *Lm* infection of macrophage cells, promoting *comK* gene expression and hence *Lm* intracellular growth.

### MpaR functions as an anti-repressor of both phage elements

**Fig. 4:**
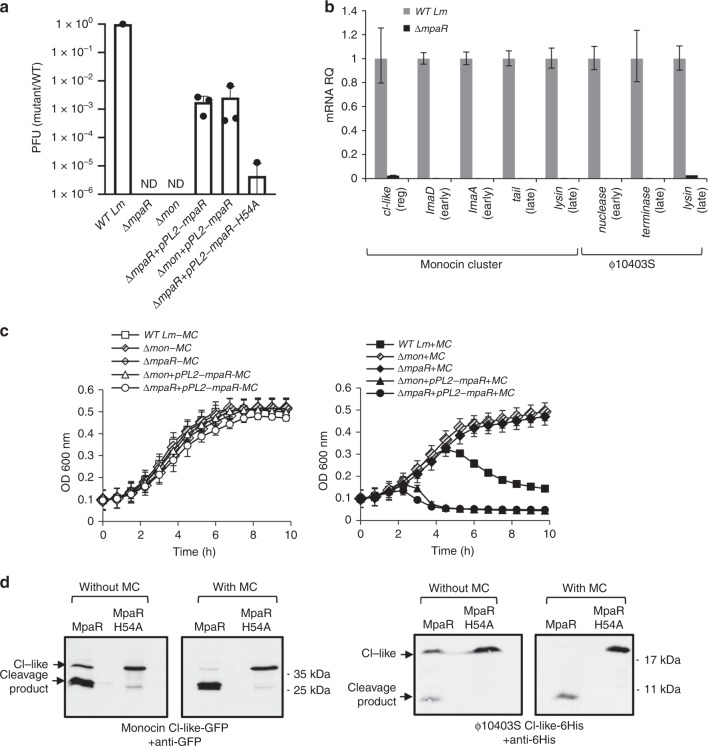
MpaR functions as an anti-repressor of both monocin and ϕ10403S. **a** A plaque forming assay of WT *Lm*, Δ*mpaR* and Δ*mon* mutants, as well as their complemented strains harboring *mpaR* gene on pPL2 (Δ*mpaR+*pPL2-*mpaR* and Δ*mon+*pPL2-*mpaR*, respectively), 4 h post MC treatment at 30˚C. Also included ∆*mpaR* mutant that was introduced with *mpaR*-H54A variant on pPL2 (Δ*mpaR+*pPL2-*mpaR*-H54A). The results are normalized to PFU of WT bacteria. Error bars represent standard deviation of 3 independent experiments. **b** qRT-PCR analysis of transcription levels of indicated genes of monocin-cluster or ϕ10403S under SOS (4 h post MC treatment at 30˚C) in WT and Δ*mpaR* bacteria. Transcription levels are represented as relative quantity (RQ), relative to their levels in WT bacteria. The data represent three independent experiments. Error bars indicate a 95% confidence interval. **c** Growth of WT *Lm*, Δ*mpaR* and Δ*mon*, as well as their complemented strains harboring the *mpaR* gene on pPL2 under the constitutive *veg* promoter (Δ*mpaR+*pPL2-*mpaR* and Δ*mon+*pPL2-*mpaR*, respectively) without (left panel) and with (right panel) MC. The data shows the mean and the standard deviation of three independent biological repeats. **d** Western blot analysis of CI-like repressors cleavage by MpaR. Δ*mon/*Δ*ϕ* bacteria harboring pPL2 expressing translational fusions of mon-CI-like-GFP (left panel) and ϕ10403S CI-like-6-His (right panel), in addition to MpaR or MpaR-H54A variant, under the *tetR* promoter. Equal amounts of total protein from bacteria grown in the presence and absence of MC were separated on 15% SDS-PAGE, blotted and probed with anti-GFP antibodies for mon-CI-like repressor or anti-6His antibodies for ϕ10403S-CI-like repressor. The experiment was performed 3 times, and the figure shows representative blots. Source data are provided as a Source Data file.

The monocin *cI-like* and *mpaR* genes were previously predicted to encode a toxin/anti-toxin system^27^. However, a close examination of their amino acid sequences revealed that they more closely resemble the classic repressor-protease protein duo, a motif found in many temperate phages and phage-derived elements. The best studied example is ImmR-ImmA in the mobile genetic element ICE*Bs*1 of *Bacillus subtilis*^28^. This protein duo comprises a regulatory mechanism in which, in response to a given signal (e.g., DNA damage), the protease (in this case, ImmA) functions as an anti-repressor, by directly cleaving its cognate repressor (ImmR), thus triggering the transcription of the downstream “lytic” genes^29^. Typically, temperate phages carry such a protease anti-repressor activity as a separate protein, encoded immediately downstream of the phage main repressor, or as a dedicated domain (a peptidase domain), which is imbedded within the repressor itself, as seen in the λ phage CI-repressor (in this case the repressor undergoes autocleavage under stress). Unexpectedly, we could not identify any gene encoding such a putative protease/peptidase activity in the ϕ10403S-genome. There were no homologs of the *mpaR* gene or other protease/peptidase genes, and the CI-like repressor (LMRG_01514) lacked a putative peptidase domain. Interestingly, by examining other related A118-like phages (of different *Listeria* strains) we found many to carry an *mpaR* homolog immediately downstream their *cI-like* repressor gene (Supplementary Table 1). During these characterizations, we noted that the originally sequenced A118 phage of strain WSLC1118 encodes a putative metalloprotease (protein Gp35) that shares high similarity with the MpaR of the monocin (62.5% similarity and 38.7% identity of amino-acid sequences). These observations led us to speculate that ϕ10403S may employ the monocin metalloprotease as an anti-repressor instead of coding for one. To address this hypothesis, we examined the ability of ϕ10403S to switch into lytic production (under SOS) in the absence of MpaR and found it incapable. No infective virions were detected in a ∆*mpaR* mutant (or in a ∆*mon* mutant), a phenotype that was complemented by providing a copy of an *mpaR* gene on the pPL2 plasmid (pPL2-*mpaR* was introduced to both ∆*mpaR* and ∆*mon* mutants) (Fig. 4a). Additional experiments indicated that ∆*mpaR* bacteria fail to induce lytic-gene transcription of either of the phage elements (shown by selected genes representing the different gene modules i.e., regulatory, early and late, Fig. 4b), and do not undergo bacterial lysis under induction of the SOS response (Fig. 4c). The results of the experiments demonstrate that MpaR plays a critical role in the de-repression (induction) of the two phage elements and moreover, that MpaR is sufficient for ϕ10403S induction since the introduction of pPL2-*mpaR* to ∆*mon* bacteria essentially complemented both virion production and bacterial lysis driven by ϕ10403S (Fig. 4a, c).

To verify the possibility that MpaR functions as the co- anti-repressor of both phage elements, we examined its ability to cleave each CI-like repressor (i.e., ϕ-CI-like and mon-CI-like) under SOS. As a control for MpaR protease activity, we constructed an MpaR variant with a mutation of histidine to alanine in the predicted metal-binding site (i.e., EELMH to EELMA), MpaR-H54A. This conserved site is known to be essential for the protease activity of the DUF955 family of metalloproteases to which MpaR belongs^26,29^. To examine whether the mutated MpaR protein is stable and expressed similarly to the native MpaR, both proteins were fused to GFP in their C’-terminus and their levels were evaluated using Western blot analysis. We found both proteins to be similarly expressed and stable (Supplementary Fig. 4). As predicted, the MpaR-H54A variant failed to complement virion production in ∆*mpaR* bacteria (using pPL2-*mpaR-H54A*, in comparison to a plasmid carrying the native *mpaR* gene), indicating that MpaR’s protease activity is required for ϕ10403S lytic induction (Fig. 4a). To assess the ability of MpaR to promote the cleavage of the two main CI-like repressors, they were first tagged with a GFP or His-tag (mon-CI-like-GFP and ϕ-CI-like-His), and were each co-expressed with native or mutated MpaR from the pPL2 plasmid (pPL2*-mon-cI-gfp-mpaR*, pPL2-*mon-cI-gfp-mpaR-H54A*, pPL2*-ϕ-cI-His-mpaR*, and pPL2-*ϕ-cI-His-mpaR-H54A*). Next, the plasmids were conjugated to a strain lacking both phage elements (∆*mon*/∆*ϕ*). MpaR-mediated cleavage of each CI-like repressor was monitored in vivo in bacteria grown in the presence or absence of MC. Anti-GFP and anti-His antibodies were used to detect the full-length repressor proteins and their cleavage products on Western blots. The results indicated that MpaR mediates the cleavage of both CI-like repressors, a phenotype that was dependent on its protease activity **(**no cleavage was observed with the MpaR-H54A variant, Fig. 4d). Of note, the MpaR activity was higher under SOS conditions, with only partial cleavage observed unless MC was added (Fig. 4d). Taken together, these findings indicate that MpaR functions as the anti-repressor of both phage elements, corroborating its aforementioned naming as a **m**etallo**p**rotease **a**nti-**r**epressor (MpaR).

### MpaR activation in macrophages is independent of the SOS response

**Fig. 5:**
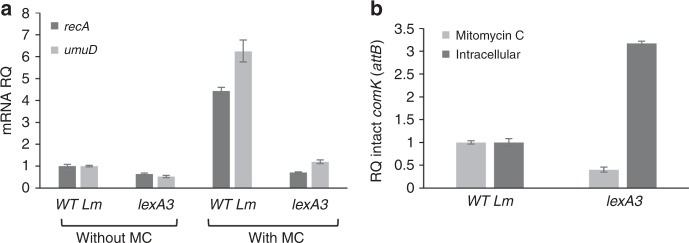
The role of the SOS response in MpaR activation. **a** qRT-PCR transcription analysis of SOS response representative genes (*recA* and *umuD*) in WT bacteria and bacteria harboring *lexA3* mutation, with and without MC treatment at 30 ˚C. Transcription levels are presented as relative quantity (RQ), relative to their levels in untreated WT bacteria. The data represents three independent experiments. Error bars indicate a 95% confidence interval. **b** qRT-PCR transcription analysis of intact *comK* gene (*attB* site) in WT bacteria and bacteria harboring the *lexA3* mutation upon MC treatment or during intracellular growth in macrophages (6 h post infection). *attB* levels are presented as relative quantity (RQ), relative to their levels in WT bacteria. The data represents three independent experiments. Error bars indicate a 95% confidence interval. Source data are provided as a Source Data file.

To examine whether the activation of MpaR under MC treatment and during *Lm* intracellular growth is mediated by the SOS response, we generated a *lexA3* mutation in *Lm* 10403S *lexA* gene that renders LexA protein uncleavable and therefore not responsive to DNA damage (glycine at position 91 was changed to aspartic acid, G91D). This *lexA3* mutation was characterized before in *E. coli* (G85D)^30^. As expected, under MC treatment *lexA3* mutant failed to activate SOS genes in comparison to WT bacteria, as shown for *recA* and *umuD*, two key genes of the SOS response (Fig. 5a). Having this mutant in hand, we next examined the excision of ϕ10403S prophage under MC treatment and during *Lm* intracellular growth in macrophage cells. Notably, we found that ϕ10403S excision under MC treatment requires the SOS response, whereas ϕ10403S excision in macrophage cells does not. In fact, an enhanced prophage excision was detected in *lexA3* bacteria grown in macrophage cells in comparison to WT bacteria (Fig. 5b). These findings suggest that MpaR is activated by a different mechanism within the phagosomes of macrophage cells, and that it is not solely responding to DNA damage.

### ϕ10403S is controlled by the monocin regulatory switch

**Fig. 6:**
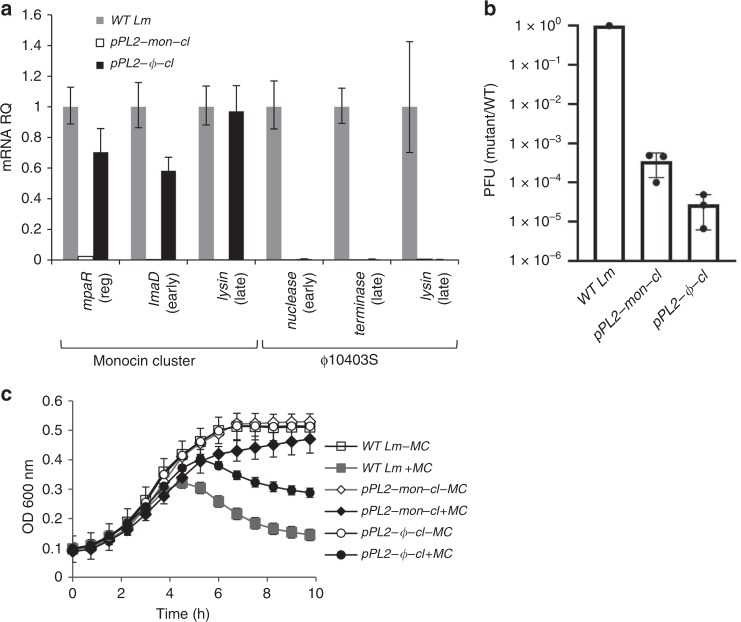
The monocin cluster CI-like repressor regulates ϕ10403S induction. **a** qRT-PCR transcription analysis of monocin cluster and ϕ10403S representative genes in WT bacteria and bacteria constitutively over-expressing mon-CI-like or ϕ-CI-like repressors from pPL2 (pPL2*-mon-cI* and pPL2*-ϕ-cI*, respectively), 4 h post MC treatment at 30 ˚C. Transcription levels are presented as relative quantity (RQ), relative to their levels in WT bacteria. The data represent 3 independent experiments. Error bars indicate a 95% confidence interval. **b** A plaque forming assay performed on WT *Lm* or bacteria constitutively over-expressing mon-CI-like or ϕ-CI-like repressors from pPL2 (pPL2*-mon-cI* and pPL2*-ϕ-cI*, respectively), 4 h post MC treatment at 30 ˚C. The results were normalized to the results with WT bacteria. Error bars represent the standard deviation of three independent experiments. **c** Growth analysis of WT *Lm* or bacteria constitutively over-expressing mon-CI-like or ϕ-CI-like repressors from pPL2 under the constitutive *veg* promoter (pPL2*-mon-cI* and pPL2*-ϕ-cI*, respectively) with or without MC. The data shows the mean and the standard deviation of three independent biological repeats. Source data are provided as a Source Data file.

The results presented so far demonstrate that MpaR has two substrates, its cognate CI-like repressor (mon-CI-like) and the ϕ10403S CI-like repressor (ϕ-CI-like), revealing the mechanism by which the lytic induction of the two phage elements is coordinated. To further explore the regulatory hierarchy between the two elements, we overexpressed each CI-like repressor in WT bacteria (using pPL2-*mon-cI* and pPL2-*ϕ-cI*) and examined their impact on the regulation of the phage elements under SOS. Interestingly, the mon-CI-like repressor could affect both elements, and ϕ-CI-like repressor affected only its cognate phage, ϕ10403S (Fig. 6a, b). Over-expression of the mon-CI-like repressor inhibited the transcription of both phage elements (as shown by considering representative genes, Fig. 6a), the production of ϕ10403S infective virions (Fig. 6b), and bacterial lysis under SOS (Fig. 6c). In contrast, overexpression of the ϕ-CI-like repressor inhibited the transcription of its cognate phage, as well as virion production, but had no effect on the transcription of the monocin or the monocin-mediated bacterial lysis under SOS (Fig. 6a–c). The results also provided evidence that the effect of the mon-repressor on ϕ10403S is likely to be indirect (mediated by MpaR), as overexpression of mon-CI-like repressor also inhibited the transcription of the *mpaR* gene, which we found to promote ϕ10403S induction (Fig. 6a). This observation indicated that the monocin repressor auto-regulates its own *repressor-metalloprotease* gene duo, as shown to be the case for ImmR/ImmA of ICE*Bs*1 and other related systems^29,31,32^. Altogether, these results establish ϕ10403S as a non-autonomous (defective) prophage that is regulated by another phage-derived element that co-inhabits the genome.

### MpaR is a global coordinator of cohabiting prophages

**Fig. 7:**
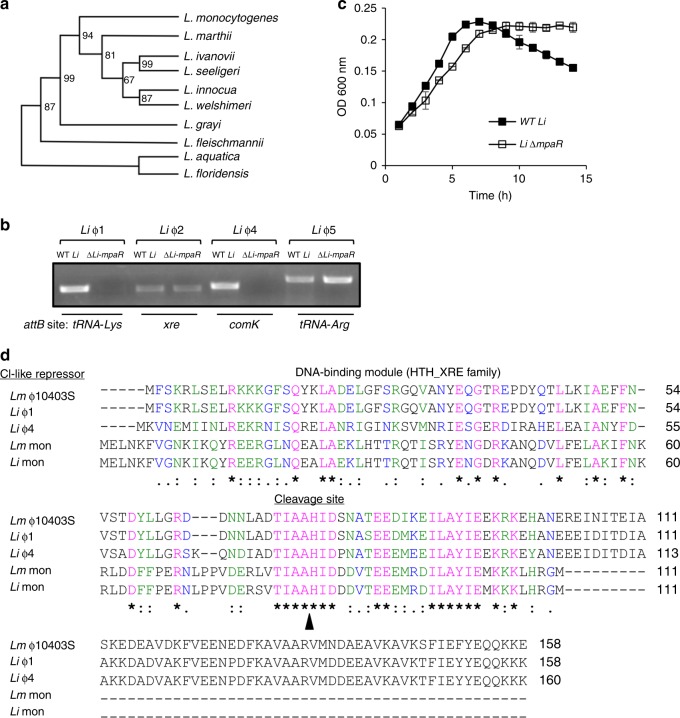
MpaR functions as a multi-phage coordinator. **a** Phylogenetic tree of *Listeria* species based on the amino acid sequence of MpaR (using 12 selected *Listeria* strains that represent the different *Listeria* species, as performed by Orsi et al. 2016). Bootstrap values are indicated on the relevant branches. **b** PCR analysis of the genomic region around the *attB* sites of *L. innocua* Clip112624 prophages, performed in wild-type bacteria (WT *Li*) and in a strain deleted of the *mpaR* gene (∆*Li-mpaR*) under MC treatment. Visible PCR products indicate phage excision and restoration of the *attB* sites. The genes harboring the prophages *attB* sites are indicated at the bottom. **c** Growth analysis of WT *L. innocua* (WT *Li*) and its isogenic *mpaR* mutant (*Li*-Δ*mpaR*) with MC (growth without MC is presented in Supplementary Fig. 6). The data shows the mean and the standard deviation of three independent biological repeats. Source data are provided as a Source Data file. **d** Multiple amino acid sequence alignment (by Clustal Omega) of the five different CI-like repressors that were found to be regulated by MpaR in *Lm* 10403S and *L. innocua* Clip112624 (*Li*). Identical amino acid residues (aa) are marked with (*), strongly similar aa are marked with (:), and weakly similar aa are marked with (.). The exact cleavage site of MpaR, as obtained by LC-MS/MS analysis of ϕ10403S CI-like repressor cleavage product, is indicated by a black arrowhead.

Intrigued by this inter-phage regulatory hierarchy, we sought to learn more about this novel interaction and analyzed the history of MpaR in *Listeria*. To this end, we constructed a phylogenetic tree based on the MpaR amino-acid sequence and compared it to a tree that was recently constructed by Orsi et al., (2016) using 325 listerial core proteins^33^. Interestingly, we found the two trees to be identical, with the exception of one monophyletic group of *Listeria* species that was lacking the *mpaR* gene, as well as the entire monocin locus (Fig. 7a). Of note, this group was recently named *Paenilisteria*, which means almost *Listeria*, as it seems to have diverged very early in the evolution of the genus^33^. This comparative phylogenetic analysis implies that the *mpaR* gene, including the monocin locus, was inserted very early, at the beginning of *Listeria* evolution, maybe even before it diverged to form a clade (if one does not consider *Paenilisteria* as *Listeria*), and was then inherited vertically. Furthermore, we found the *mpaR* gene and the entire monocin locus to be highly conserved in all *Listeria* species (with the exception of *Paenilisteria*). Of note, we identified similar bacteriocin clusters in some *Enterococcus* and *Streptococcus* strains. In *Listeria* we found the monocin locus to contain a maximum of 17 genes with some exceptional *Listeria* strains harboring shorter versions of 3–6 genes, all including the *mpaR* gene (Supplementary Fig. 5). In light of these observations we speculated that MpaR may play a more fundamental role in *Listeria*, possibly functioning as a global coordinator of cohabiting prophages and phage-derived elements, co-regulating their lytic induction under stress. To explore this hypothesis, we chose the non-pathogenic *L. innocua* strain CLIP 11262 (named here *L. innocua* or *Li* in short), since we found its genome to carry 5 different prophages in addition to the monocin (named here *Li*-ϕ1 to *Li*-ϕ5, and *Li*-monocin, respectively Table 1). Notably, the monocin locus of *L. innocua* CLIP 11262 and *L. monocytogenes* 10403S are very similar, exhibiting 85% identity of DNA sequence, with 98% similarity in the amino-acid sequence of the MpaR proteins. Genomic analysis of *L. innocua* prophages revealed that except for *Li*-ϕ2, they all resemble known *Listeria*-specific phages (A118, A500, B054, and A006, Table 1). The ability of each prophage to excise the genome under SOS was evaluated by using PCR to monitor the re-formation of the relevant *attB* site (Table 1). Genomic excision in the presence of MC was detected in four of the prophages, *Li*-ϕ1, ϕ2, ϕ4, and ϕ5, while *Li*-ϕ3, remained integrated within the bacterial chromosome (Table 1). To examine whether *L. innocua* MpaR plays a role in the regulation of the excised prophages, *i.e*., controlling their genomic excision and lytic induction, we generated an *mpaR* deletion mutant of *L. innocua* (∆*Li-mpaR*) and tested for prophage excision under SOS in the mutant compared to WT *Li* bacteria. We found that the MpaR of *L. innocua* controls the excision of *Li*-ϕ1 and *Li*-ϕ4 (Fig. 7b), suggesting that it regulates at least 3 distinct phage elements that inhabit the chromosome (if including its own encoding monocin element). Remarkably, both the *Li*-ϕ1 and *Li*-ϕ4 genomes lack an *mpaR* homologue (as observed in ϕ-10403S), whereas *Li*-ϕ2 and *Li*-ϕ5 carry a metalloprotease gene immediately downstream of their CI-like repressor gene (Table 1). That said, examining the growth of ∆*Li-mpaR* mutant under MC treatment we found it doesn’t undergo bacterial lysis, while WT *L. innocua* does (Fig. 7c and Supplementary Fig. 6), overall indicating that the MpaR of *L. innocua* essentially controls all the lytic prophages that inhabit the chromosome, a phenotype that is similar to the MpaR of *Lm* 10403S. In this regard, it is possible that *Li*-ϕ2 and *Li*-ϕ5 are defective prophages, since they fail to trigger bacterial lysis despite their genomic excision under SOS.

**Table 1:** Phage elements in *L. innocua* CLIP 11262.

Prophage	*attB* site	Genome length	Phage orthocluster	Contains MpaR?	Excision upon SOS
*Li* ⏀1	*tRNA-Lys*	39.4 kb	A500-like	No	Yes
*Li* ⏀2	Xre family regulator	50.56 kb	–	Yes	Yes
*Li* ⏀3	*tsf*	49.2 kb	B054-like	Yes	No
*Li* ⏀4	*comK*	39.3 kb	A118-like	No	Yes
*Li* ⏀5	*tRNA-Arg*	38.4 kb	A006-like	Yes	Yes
*Li*-monocin	Between *lin0159* and *lin0177*	10.7 kb	Monocin	Yes	No

Names of genes and species are in italics

Having established that MpaR controls the lytic response of cohabiting phage elements in both *L. innocua* CLIP 11262 and *L. monocytogenes* 10403S strains, we compared the protein sequence of all of the elements CI-like repressors, which are potential substrates for MpaR (5 repressors in total, of ϕ10403S, *Li*-ϕ1, *Li*-ϕ4, *Lm*-mon, and *Li*-mon), and searched for a common MpaR recognition or cleavage site. As shown in Fig. 7d, we identified a single region (TIAAHIDxxxxEExxxxILAYIE) that is conserved in all the repressors located downstream to their HTH-DNA binding domain. To examine whether this conserved region represents the cleavage site of MpaR, we used mass-spectrometry (LC-MS/MS) to analyze the cleavage products of the ϕ-CI-like repressor (shown in Fig. 4d) and discovered that it was cleaved precisely between the alanine and histidine of the TIAAHID sequence (between A_74_ and H_75_). Interestingly, systematic analysis of *comK*-phages in all available and complete *Listeria* genomes revealed 20 more prophages (of different *Listeria* strains) that lack an *mpaR* homolog and that contain the TIAAHID sequence in their CI-like repressors (Supplementary Table 1). Altogether, these observations strengthen the premise that MpaR has been coopted to play a central role in *Listeria*, functioning as a **m**ulti-**p**hage **a**nti-**r**epressor (also MpaR) that coordinates the lytic induction of cohabiting phage elements, whether infective or defective. In addition, the results indicate that co-regulation of resident prophages is a common feature in *Listeria*, a function that most likely evolved to synchronize their responses with the various host life styles.

## Discussion

Polylysogeny, the carriage of multiple prophages and phage-derived elements, is a widespread phenomenon in bacteria, yet the governing mechanisms that maintain this intricate interaction remain unclear^1^. It is generally considered to be the result of a gradual evolutionary process, in which bacteria frequently encounter lysogenic phages that integrate their genome into the bacterial chromosome. It is therefore reasonable to speculate that each integration event would provoke a new round of a multi-front battle, in which the newly incoming phage competes not only with its bacterial host, but also with the existing pool of resident phage elements for the successful production of infective virions. Evidently, during this struggle some prophages lose (being neutralized^34,35^), while others succeed in remaining active (sometimes using innovative ways as exemplified here), and produce progeny virions that are released to the environment. That said, polylysogeny is also considered an adaptive process for the host, as prophages that stably inhabit the chromosome (infective and defective) were shown to contribute to the fitness of their host by supplying new genes and functions (e.g., virulence, metabolic, warfare, and defense)^3,36,37^. These observations indicate that polylysogeny is a highly dynamic evolutionary process in which unusual and innovative inter-phage and bacteria–phage interactions can develop that promote bacteria–phage cooperation under certain circumstances^14^. In this study we provide a molecular insight into one such example of a polylysogenic interaction between *Lm* strain 10403S and its two-cohabiting phage-elements, the first producing infective virions (ϕ10403S) that serves as a molecular switch within mammalian cells, and the other cryptic (monocin) producing bacteriocins under SOS. Our results demonstrate that the two phage elements are essentially synchronized and perfectly adapted to the intracellular life style of their host. They exhibit a regulatory hierarchy in which the evolutionarily more ancient cryptic element controls the induction of the active phage. The data provide evidence for a model in which a metalloprotease (MpaR), encoded by the cryptic phage element has been co-opted to function as a multi-phage anti-repressor that is induced under conditions of stress (SOS or in response to stresses encountered in mammalian cells) to coordinate the lytic induction of cohabiting phage elements (infective and defective). This mechanism may well have evolved to align the responses of resident phage elemnets with the interests of the host, promoting their mutual survival in a changing environment. The findings further suggest that regulatory factors from phage origin, which are ubiquitous in bacterial genomes, can be exploited by bacteria to control newly incoming phages, thereby easing their adaptation to the host. Moreover, they also point out a strategy by which newly incoming phages can retain their activity by hijacking the regulatory factors of more ancient resident phage elements that have already adapted to the host, aligning their responses in order to increase their fitness. Altogether, this study demonstrates that cryptic phage elements play an important role in the interaction between bacteria and lysogenic phages, contributing to the development of mutually beneficial relationships.

Here we show that the two phage elements of *Lm* 10403S strain are active, concomitantly producing infective virions and monocins under SOS. We found the monocins to be functional, capable of killing assorted *Listeria* strains of different species, representing a classic example of bacterial domestication of phage components^36,38^. The results of this study revealed that the lysis modules of the two elements are equally potent and redundant, overall demonstrating that their lytic production is synchronized. In this regard, it makes sense for the phage to synchronize its lytic production with that of the monocin, as an early bacterial lysis driven by the monocin can hinder the formation of infective virions. This synchronization further raises the question of whether the particles themselves (i.e., the monocins and virions) may interact with each other and jointly influence (enhance) bacterial fitness under stress. Bacteriocins are known to provide a competitive growth advantage to their host by killing neighboring target cells^39^. As bacteriocins release requires bacterial lysis, only part of the population produces bacteriocins for the benefit of others, as a form of altruism. In a way, infective prophages act in a similar manner, where lytic-production is initiated only in part of the population and target cells are killed via lytic infection. Several studies have indicated that temperate phages can provide their bacterial host with a growth advantage by killing neighboring non-lysogenic cells^40–42^. Therefore, it is possible that monocins and infective virions cooperatively enhance *Lm* competitiveness in communal environments, providing a possible explanation for the evolutionary linkage we uncovered here. That said, the observation that the two phage-elements maintain two functionally redundant lysis modules suggests they might be differentially regulated under certain conditions, e.g., in the extracellular environment or in other natural hosts, as otherwise they could potentially share one of them.

An unexpected finding in the study was that the monocin encoded MpaR, in both the *Lm* 10403S and *L. innocua* CLIP 11262 strains, can serve as a global anti-repressor of lytic phage elements, infective and defective. Remarkably, none of the elements possessed their own *mpaR* homolog or functional analog within their genome, and were completely dependent on host MpaR for re-activation. In the *Lm* 10403S, MpaR was responsible for de-repressing both phage elements under SOS and intracellular growth, however during infection of macrophage cells this function was not dependent on the SOS response. These findings suggest that there is another stress signal within the macrophage phagosomes that triggers the activation of MpaR and therefore ϕ10403S excision from *comK*. Notably, this ϕ10403S excision from *comK* does not lead to lytic production (of both monocins and phage virions), thus MpaR essentially functions as the main regulator of *comK* gene expression in macrophage cells. These findings relates to our previous finding that ϕ10403S excision in mammalian cells enables the expression of *comK*^12^, thereby promoting the expression of the *com* genes, some of which facilitate bacterial escape from the phagosomes and thus contribute to bacterial virulence. We further demonstrate that this adaptive function of the phage elements relies on the repression of their lytic pathway and specifically the lysis genes, thus enabling ϕ10403S to act as a regulatory DNA element (exhibiting a high rate of re-integration) without killing the host within the intercellular niche. We found the monocin structural and lysis genes to be strongly repressed during *Lm* intracellular growth, implying the existence of a mechanism downstream of MpaR that specifically represses the lytic genes in the course of *Lm* infection of macrophage cells. Altogether, the results presented here demonstrate a bacteria–phage co-adaptation, in which cohabiting phage elements cooperatively form an orchestrated molecular switch that regulates bacterial gene expression to promote virulence.

Pirating phage-derived structural and regulatory determinants is a well-documented phenomenon, although most cases involve mobile genetic elements. The best example is given by *Staphylococcus aureus* pathogenicity islands (SaPIs), which inhabit many staphylococci and encode for virulence factors^43^. These SaPIs passively reside in the chromosome, and remain repressed by their own repressor StI, until they are excised by infection or induction of a helper prophage. They then replicate and are packaged into phage-like particles that carry them to a new host. De-repression of the SaPIs requires the expression of an anti-repressor encoded by the helper phage (which is non-essential for the phage), that binds the StI repressor, causing it to dissociate from the SaPI DNA^44,45^. Interestingly, Lemire et al., have demonstrated that this non-classical mechanism of de-repression (as opposed to the classical method of repressor proteolysis) is also found in *Salmonella* prophages, which encode for small proteins that function as anti-repressors, via direct binding to their cognate repressors^46^. Notably, they also demonstrated that these prophages, e.g., Gifsy-1 and Gifsy-3, which commonly inhabit the chromosomes of polylysogenic *Salmonella enterica* strains, respond to one another’s anti-repressor, thereby synchronizing their lytic induction upon SOS. Another interesting mechanism that is used to co-regulate prophages and other latterly transferred DNA elements is xenogeneic silencing^47^. Bacteria often encode for proteins that function as global silencers of prophages, thus promoting the tolerance of phage DNA. Recently, such a phage encoded protein was identified in *Corynebacterium glutamicum* (CgpS) that functions as xenogeneic silencer of prophages and other foreign DNA elements via binding to AT-rich DNA sequences^48^. Overall, these findings strengthen the premise that coordination of cohabiting prophages is a common feature of bacteria. In addition, they provide a molecular insight into the evolutionary path by which the sort of inter-phage dependency seen for *Lm* ϕ10403S and *L. innocua* ϕ1 and ϕ4, depending on the monocin, might evolved. It is possible that MpaR first evolved to recognize the repressors of its neighboring prophages and coordinate their lytic responses, and only later did the prophages lose their own anti-repressor genes via natural selection. While this process renders the prophages non-autonomous (i.e., defective), they are still active (producing infective virions), as they can still propagate via the lytic cycle, which does not require the lysogenic-lytic switch for virion production (demonstrated for ϕ10403S infecting ∆*mon/*∆*ϕ* bacteria, Supplementary Fig. 7). Moreover, since MpaR is widely spread and highly conserved in *Listeria* species, it is most likely that these phages can still lysogenize into different *Listerial* strains and remain effectively active. Overall, this study emphasizes that there are un-limited types of bacteria–phage interactions that go beyond the classic infective**/**defective phage definitions. Lysogenic phages can acquire sophisticated mechanisms to remain active/infective, even in the price of being dependent on their host (i.e., being non-autonomous prophages). This type of dependency is a result of a bacteria–phage co-adaptation that provides an advantage for both the bacteria and the phage under certain circumstances, such as within the mammalian niche. Finally, this study suggests that MpaR was domesticated to function as a multi-phage anti-repressor, representing the first example of a global coordinator of cohabiting prophages, that acts to promote bacteria–phage coexistence under complex environments.

## Methods

### Ethics statement

The use of animals in this study was limited to preparation of bone marrow derived macrophages from mice. Experimental protocols were approved by the Tel Aviv university Animal Care and Use Committee (04–18–028) according to the Israel Welfare Law (1994) and the National Research Council guide (Guide for the Care and Use of Laboratory Animals 2010).

### Bacterial strains, plasmids, and growth conditions

*L. monocytogenes* strain 10403S was obtained from Prof. Daniel Portnoy (University of California, Berkeley) and was used as a WT strain and as a parental strain of all the mutants generated in this study except when specifically indicated. *E. coli* XL-1 Blue (Stratagene) was utilized for vector propagation. *Lm* 10403S phage cured strain (DPL-4056, named here ∆*ϕ*) was generated by Prof. Richard Calendar via biological curing. *E. coli* SM-10 (BCCM collection of micro-organisms) was utilized for conjugative plasmid delivery to *Lm* bacteria. *Listeria* strains were grown in brain heart infusion (BHI) (Merck) medium at 37 °C or 30 °C as specified, and *E. coli* strains were grown in Luria-Bertani (LB) (Acumedia) medium at 37 °C. Phusion polymerase was used for all cloning purposes and *Taq* polymerase for verifications of the different plasmids and strains by PCR. Antibiotics were used as follows: chloramphenicol (Cm), 10 µg/ml; streptomycin (Strep), 100 µg/ml; and kanamycin (Km), 30 µg/ml; mitomycin C (MC) (Sigma), 1.5 µg/ml. All restriction enzymes were purchased from New England BioLabs.

### Bacterial lysis assay

Bacteria were grown overnight (O.N.) at 37 °C with agitation in BHI broth, and then the culture was diluted to an OD at 600 nm (OD_600_) of 0.15, and pipetted in triplicates into a 96-well plate with or without MC (1.5 µg/ml). The plates were incubated at 30 °C in a Synergy HT BioTek plate reader, and the OD_600_ was measured every 15 min proceeded by 2 min of shaking. All experiments were repeated at least three times.

### Plaque forming assay

Bacteria were grown O.N. at 37 °C with agitation in BHI broth, then the culture was diluted by factor of 10 in fresh BHI, incubated without agitation at 30 °C to reach OD_600_ of 0.4, diluted to an OD_600_ of 0.15, and then a lytic cycle was induced by the addition of MC (1.5 µg/ml) and incubation for 4 h. Bacterial cultures were filtered through 0.22 μm filters that do not allow the passage of bacteria. An appropriate dilution of the filtered supernatants (100 μl) were added to 3 ml of melted LB-0.7% agar medium at 56 °C supplemented with 10 mM CaCl_2_, and 300 μl of an O.N. culture of *L.* *monocytogenes* Mack861 bacteria, used as an indicator strain, and quickly overlaid on BHI-agar plates. Plates were incubated for 3–4 days at room temperature to allow plaques to form.

### Monocin killing assay

Bacteria were grown at 37 °C O.N. with agitation in BHI broth, then the culture was diluted 1:10 in BHI, incubated without agitation at 30 °C to reach an OD_600_ of 0.4, diluted to an OD_600_ of 0.15, and then a lytic cycle was induced by the addition of MC (1.5 µg/ml) and incubation for 4 h. Bacterial cultures were filtered through 0.22 μm filters. LB-0.7% agar medium melted at 56 °C (8 ml) were supplemented with 50 μl of O.N. culture of the bacterial strain of interest and quickly overlaid on 12 cm × 12 cm square BHI-agar plates. Filtered supernatants were serially diluted 1:5, and 5 µl droplets of each dilution were then plated on the bacterial lawn soft agar overlay^22^. The plates were incubated at 30 °C for 1–2 days.

### Generation of mutants, complemented and overexpressing strains

To prepare gene deletion mutants, upstream and downstream regions of the selected gene were amplified using Phusion DNA polymerase and cloned into the pLR16 vector^24^. Cloned plasmids were verified by PCR and their inserts were sequenced, then the plasmids were conjugated to *L. monocytogenes* or *L. innocua* using *E. coli* SM-10 strain. *Trans*conjugants were selected on BHI agar plates supplemented with chloramphenicol and streptomycin, and transferred to BHI supplemented with chloramphenicol for two days at 41 °C to allow plasmid integration into the bacterial chromosome by homologous recombination. The bacteria were then grown O.N. in fresh BHI medium without chloramphenicol at 30 °C to promote plasmid curing and the generation of an in-frame gene deletion. After this time, the bacteria were plated on BHI plates supplemented with p-Cl-phe (4-Chloro-L-phenylalanine, Acros) and the resistant colonies were validated for gene deletion by PCR. Complemented strains of deletion mutant were generated by introducing a copy of the intact *comK* gene *in trans* under the control of its native promoter or *mpa R*gene under the control of constitutive *veg* or inducible TetR promoter using the pPL2 integrative vector^49^. For overexpression of CI proteins, the corresponding gene was cloned into the pPL2 integrative vector and transcribed by a strong constitutive promoter (P_const_, promoter origins described at Argov et al.^24^). The pKSV7 (oriT)^50^ plasmid harboring the erythromycin resistance gene for selection in Gram-positive bacteria was used for construction of the deletion mutant in *L. innocua*, and the *trans*conjugants were selected using ceftriaxone (0.5 µg/ml) and erythromycin (1 µg/ml).

### Quantitative real-time PCR analysis

Bacteria were grown overnight at 37 °C O.N. with agitation in BHI broth, then the culture was diluted 1:10 in BHI, incubated without agitation at 30 °C to reach an OD_600_ of 0.4, was diluted to an OD_600_ of 0.15, and then a lytic cycle was induced by addition of MC (1.5 µg/ml) or UV exposure (4000 joule/cm^2^) for 4 h. Total nucleic acids were isolated by standard phenol-chloroform extraction methods where 0.04 ng of total nucleic acids were used for analysis of *attB* levels by qRT-PCR using bacterial 16S rRNA gene as a reference for sample normalization. For gene expression analysis, the samples were treated with DnaseI, and 1 μg of RNA was reverse transcribed to cDNA using a qScript (Quanta) kit. qRT-PCR was performed on 10 ng of cDNA. The relative expression of bacterial genes was determined by a comparison of their transcript levels with those of the bacterial 16 S rRNA or *rpoD* gene, which served as a reference. All qRT-PCR analyses were performed using FastStart Universal SYBR Green Master Mix (Roche) on the StepOnePlus RT-PCR system (Applied Biosystems) by the standard ∆∆C_t_ method. Statistical analysis was performed using StepOne V2.1 software. Error bars represent the 95% confidence interval.

### Transcription analysis of intracellular bacteria

BMDM cells used for infection experiments were isolated from 6 to 8 week-old female C57BL/6 mice (Envigo, Israel) and cultured in Dulbecco’s Modified Eagle Medium (DMEM)-based media supplemented with 20% fetal bovine serum, sodium pyruvate (1 mM), L-glutamine (2 mM), β-Mercaptoethanol (0.05 mM) and monocyte-colony stimulating factor (M-CSF, L929-conditioned medium); BMDM medium^51^. WT *L. monocytogenes* bacteria were used to infect BMDM seeded in a 145 mm dish, resulting in a multiplicity of infection (MOI) of ~100. Thirty minutes after infection, BMDM monolayers were washed twice with PBS to remove unattached bacteria and fresh medium was added. At 1 h post-infection (h.p.i.), gentamicin (50 µg/ml) was added to limit extracellular bacterial growth, then 6 h post infection, intracellular bacteria were liberated from the macrophages by washing with 20 ml ice cold H_2_O (RNAse free-DEPC treated), collected by passing the medium through 0.45 μM filter membranes and flash-frozen in liquid nitrogen. Bacteria were recovered from the filters by vortexing into AE buffer (50 mM NaOAc pH 5.2, 10 mM EDTA), and bacterial nucleic acids were extracted using hot (65 °C) phenol with 1% SDS followed by ethanol precipitation^52^. Rneasy Mini Kit Dnase on column (Qiagen) was used for Dnase treatment. Transcription levels of genes of interest in total RNA samples were measured with specific probes using the NanoString nCounter system, according to the manufacturer’s standard procedures^53^. Total RNA extracted from bacteria grown in BHI was analyzed in parallel as a control.

### *L.* *monocytogenes* intracellular growth

To assess the intracellular growth of *L.* *monocytogenes*, 2×10^6^ BMDM cells were seeded in a 60 mm Petri dish on glass coverslips in 5 ml of BMDM medium and incubated O.N. in a 37 °C, 5% CO_2_ forced-air incubator. *L.* *monocytogenes* were grown O.N. at 30 °C without agitation and 8×10^6^ bacteria were used to infect BMDM cells (MOI of 1). Thirty minutes post-infection, the macrophage monolayers were washed and fresh medium was added. Gentamicin was added 1 h.p.i to a final concentration of 5 μg/ml in order to limit the growth of extracellular bacteria. At each time point, three coverslips were transferred into 2 ml of sterile water to release the intracellular bacteria. Serial dilutions of the resulting lysate were plated on BHI agar plates and the CFUs were counted after 24 h incubation at 37 °C. Each of the experiments was repeated at least three times.

### *L. monocytogenes* phagosomal escape assay

1 × 10^6^ macrophage cells were seeded on 20 mm coverslip slides in BMDM medium, incubated O.N., and infected with 2 × 10^6^ *L. monocytogenes* bacteria. Thirty min post-infection, macrophage monolayers were washed twice with PBS and fresh medium was added. At 1 h.p.i. the medium was supplemented with gentamicin (50 μg/ml) to limit bacterial extracellular growth. Cells were fixed at 2.5 h.p.i. with a fixative buffer (3.7% paraformaldehyde solution), and permeabilized with 0.05% triton. Slides were then washed and stained as follows: bacteria were stained with anti-listeria-FITC antibody (Bio-Rad); actin was stained with rhodamine-phalloidin (Biotium); and DNA was stained with DAPI containing Vectashield^®^ mounting media. Images were taken using Nikon eclipse Ti-E microscope. About 200 bacteria in 4–5 different frames were counted and the statistical analysis was performed using a studentʼs t-test.

### Evaluation of ϕ10403S re-integration into *comK* gene

To assess the prophage re-integration into *comK* and the possible occurrence of phage-curing during *Lm* intracellular growth, the pheS* gene was cloned into ϕ10403S-genome (immediately downstream to *LMRG_01556* gene) as a counter-selection marker. This counter-selection system was previously shown to work in *Listeria*^24^, which uses the mutated phenylalanyl-tRNA synthetase, PheS*, that incorporates the toxic *p*-chloro-phenylalanine (*p*-Cl-phe, Acros) analog during bacterial growth. Bacteria expressing the pheS* gene fail to grow on BHI agar plates that contain 18 mM of *p*-Cl-phe. Intracellularly grown bacteria were harvested at 6 h post infection and plated on BHI agar plates with and without *p*-Cl-phe. Only bacteria that lost the phage could grow on the *p*-Cl-phe plates. Similar assays were performed on MC treated and stationary grown bacteria (at 4 h).

### Western blot analysis

The mon-CI-like repressor was tagged by translational fusion of GFP to the C’ terminus of the protein under the regulation of a constitutive promoter, while the ϕ-CI-like repressor was tagged by 6-His at C’ terminus. Both tagged CI-like repressors were cloned on the integrative pPL2 plasmid harboring the MpaR protease, and the mutated variant H54A, under the regulation of inducible *tetR* promoter, and the resulted plasmids (pPL2-*mon-cI-gfp*-*mpaR*, pPL2-*mon-cI-gfp*-*mpaR-H54A* and pPL2-*ϕ-cI-His*-*mpaR*, pPL2-*ϕ-cI-His*-*mpaR-H54A*, respectively) were delivered by conjugation into *∆mon/∆ϕ L.* *monocytogenes* bacteria. The strains were grown at 30 °C in 20 ml BHI supplemented with 100 ng/ml of anhydrotetracycline to an OD600 of 0.3. The cultures were then supplemented with MC (1.5 µg/ml), grown for an additional 2.5 h, harvested, and washed with Buffer-A (20 mM Tris-HCl pH = 8, 0.5 M NaCl, and 1 mM EDTA), resuspended in 1 ml of Buffer-A supplemented with 1 mM PMSF, and lysed by ultra-sonication. Total protein content was assayed using modified a Lowry assay, and samples with equal amounts of total proteins were separated on 15% SDS-polyacrylamide gels and transferred to nitrocellulose membranes. Proteins were probed with rabbit anti-GFP antibody (BioLegend 902601) used at a 1:5000 dilution and mouse anti-6His tag antibody (Abcam ab18184) used at a 1:1000 dilution, for detection of mon-CI-GFP and ϕ-CI-6H repressors, respectively, followed by HRP-conjugated goat anti-mouse IgG (Jackson ImmunoResearch, USA) at a 1:20,000 dilution. Western blots were developed by homemade enhanced chemiluminescence reaction (ECL). Images were obtained using Amersham imager 600 (GE Healthcare Life Siences).

### Determination of ϕ-CI-like repressor cleavage site

*∆mon/∆ϕ L.* *monocytogenes* bacteria harboring the 6his-tagged ϕ-CI-like repressor under the regulation of a constitutive promoter and the MpaR protease under the regulation of inducible *tetR* promoter on the integrative pPL2 plasmid (pPL2 P_const_ ϕ-*cI-6H* P_tetR_-*mpaR*) were grown at 30 °C in 500 ml BHI supplemented with 100 ng/ml of anhydrotetracycline to an OD600 of 0.3. The cultures were then supplemented with MC (1.5 µg/ml), grown for an additional 2.5 h, then harvested and washed with PBS. Bacteria were resuspended in 20 ml of Buffer-P (0.3 M NaCl, 50 mM NaH_2_PO_4_, pH 8) supplemented with 1 mM PMSF and 10 mM imidazole and lysed by an ultra-high-pressure homogenizer (Stansted Fluid Power) at 12,000 psi. Cell debris was removed by centrifugation at 16,000 × *g* for 20 min and the lysate was incubated with 0.5 ml of Ni-NTA beads (Sigma®) for 1 h at 4 °C with tilting. The Ni-NTA beads were then loaded on a column and washed with 10 ml of Buffer-P supplemented with 10 mM imidazole and then with 25 mM imidazole. The protein was eluted by 250 mM of imidazole in Buffer-P and dialyzed against 100 ml of Buffer-P. Eluted fractions were separated on 15% SDS-polyacrylamide gels and stained with Coomassie brilliant blue to yield two primary visible bands: of full-length ϕ -CI-like and its cleavage product. The stained band representing the cleavage product was isolated from the gel and analyzed by peptide mass fingerprinting at The Smoler Protein Research Center at the Technion, Haifa, Israel. Protein samples were digested by trypsin, and the resulting proteolytic peptides were analyzed by LC-MS/MS on Orbitrap XL (Thermo) and identified by Discoverer software version 1.4.

### Phylogenetic tree

A schematic of the phylogenetic history of the genus *Listeria* is published in Orsi and Wiedmann (2016)^33^. MpaR protein sequence was obtained from NCBI for each strain used (*L. fleischmannii* FSL S10–1203, *L. aquatica* FSL S10–1188, *L. floridensis* FSL S10–1187, *L. monocytogenes* 101403S, *L. marthii* FSL S4–120, *L. innocua* Clip11262, *L. welshimeri* serovar 6b str. SLCC5334, *L. ivanovii* WSLC3009, *L. seeligeri* FSL S4–171, *L. grayi* DSM 20601) by BLAST, and the sequences were aligned using the webPRANK server of the European Bioinformatics Institute (https://www.ebi.ac.uk/goldman-srv/webprank/). The .msa file was converted to PHYLIP format using webPRANK server and the tree assembly was obtained using ATGC:PhyML server (http://www.atgc-montpellier.fr/phyml/).

### Statistical analysis

All data with the exception of intracellular growth curves (Figs. 1d, 2a, 2d and 3c) are presented as mean ± 1 standard deviation. Replicate values are in triplicates unless indicated otherwise. For each intracellular growth experiment 3 to 6 biological repeats are shown separately in the figures and in the source file.

### Reporting summary

Further information on research design is available in the Nature Research Reporting Summary linked to this article.

## Supplementary information

Supplementary Information

Reporting Summary

Source Data

## Data Availability

Source data underlying Figs. 1a–e, 2a–d, 3a–d, 4a–d, 5a–b, 6a–c and 7c, and Supplementary Figs, 1, 2, 4, 6 and 7, as well as the list of primers used in this study, are provided as a Source Data file. The authors declare that all data supporting the findings of this study are available within the paper and its Supplementary Information files or from the corresponding author upon request.
