# Supplementary material for: Coordination of cohabiting phage elements supports bacteria–phage cooperation
Source: Nat Commun. 2019 Nov 21;10:5288. doi: 10.1038/s41467-019-13296-x (PMC6872733; doi:10.1038/s41467-019-13296-x)
Supplement: Supplementary file 1 — Supplementary Information [file 41467_2019_13296_MOESM1_ESM.pdf]

Supplementary information.

Coordination of cohabiting phage elements supports bacteria-phage cooperation.

Argov et al.

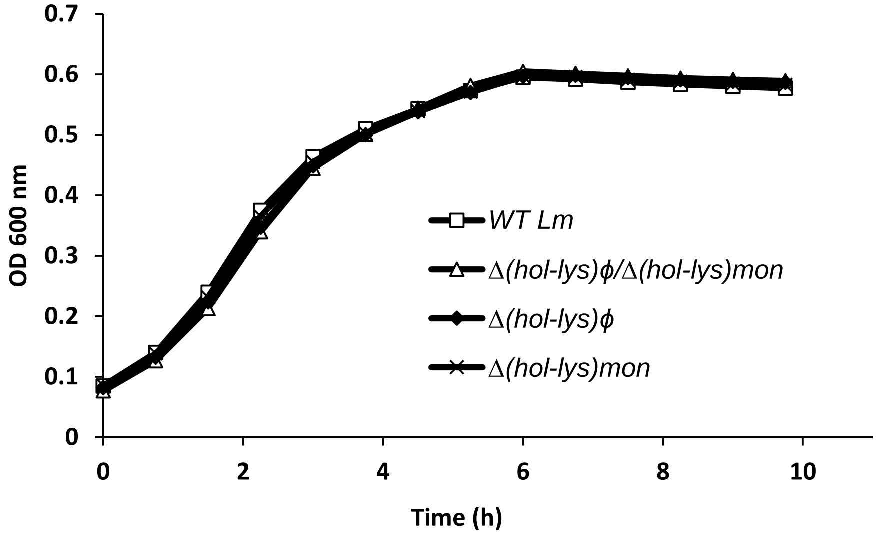

Supplementary Figure 1. Growth analysis of WT *Lm* and lysis modules mutants in the absence of MC. Growth analysis of WT *L. monocytogenes* (*Lm*) and mutants harboring deletions of the phage elements lysis modules; *LMRG_01552-4* of ϕ10403S (*Δ(hol-lys)$_{\phi}$*), and *LMRG_02377-8* of monocin cluster (*Δ(hol-lys)$_{mon}$*), or a mutant deleted of both lysis modules (*Δhol-lys)$_{\phi}$ /Δ(hol-lys)$_{mon}$*), without MC. Complementary to **Fig 1A**. The data shows the mean and the standard deviation of three independent biological repeats. Source data are provided as a Source Data file.

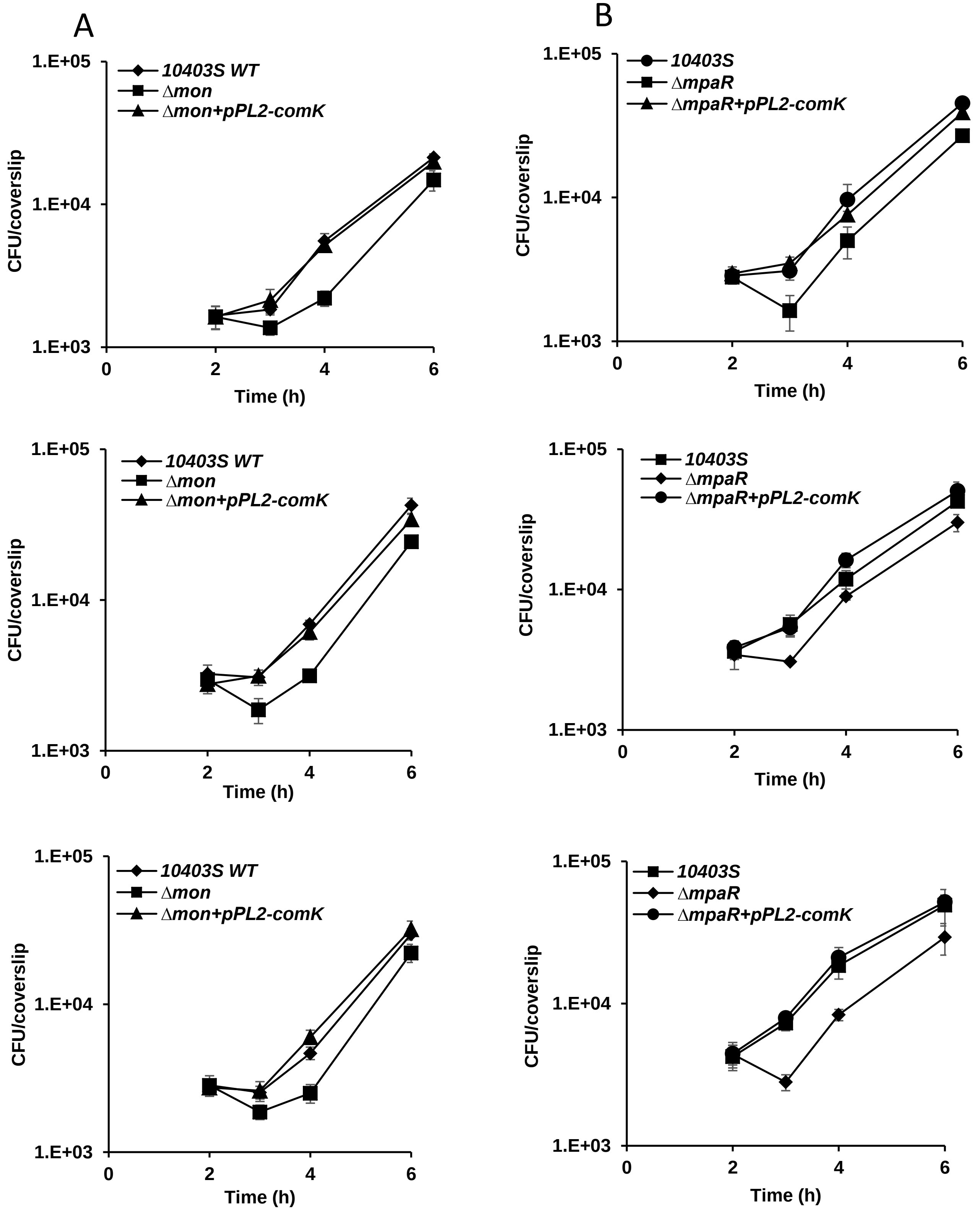

Supplementary Figure 2. Intracellular growth analysis of Δ*mon* and Δ*mpaR* mutants and their complemented strains in BMDM cells. A. Intracellular growth analysis of WT *Lm*, mutant lacking monocin cluster (Δ*mon*) and its complemented strain (Δ*mon+pPL2-comK*). **B**. Intracellular growth analysis of WT *Lm*, mutant lacking MpaR (Δ*mpaR*) and its complemented strain (Δ*mpaR+pPL2-comK*). Each graph represents one biological repeat. Three biological repeats are shown for each experiment supporting data presented in **Figs 2D and 3C**. Error bars represent standard deviation for triplicate samples. Source data are provided as a Source Data file.

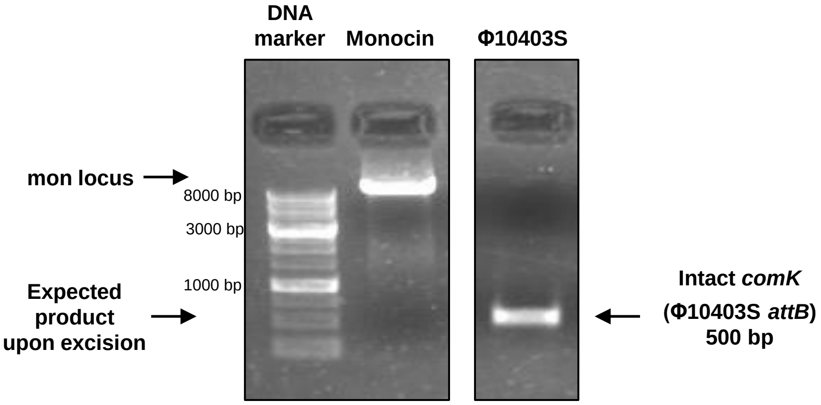

Supplementary Figure 3. The monocin does not undergo genomic excision. PCR analysis of the genomic region around the monocin locus, performed on WT *Lm* bacteria grown with MC (4h post MC treatment at 30˚C). As a control excision of ϕ10403S is shown.

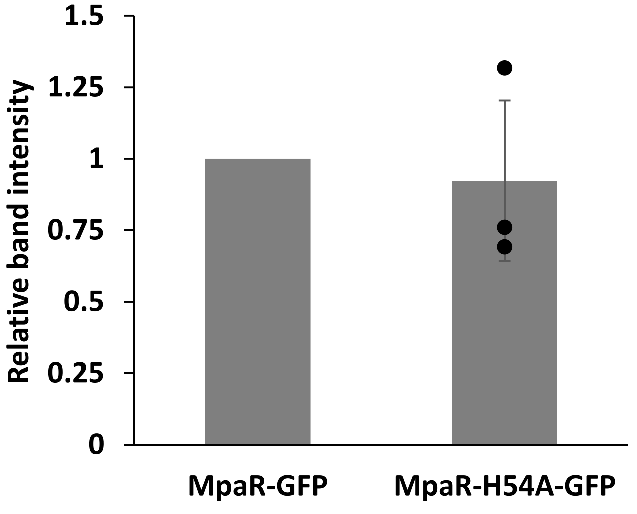

Supplementary Figure 4. MpaR and its H54A variant are similarly expressed. Quantitation of Western blot analysis of GFP-tagged MpaR (MpaR-GFP) and its H54A variant (MpaR-H54A-GFP), probed with anti-GFP antibody. The MpaR repressor was tagged by translational fusion of GFP to the C' terminus of the protein under the regulation of the *tetR* promoter, and the resulted plasmids (pPL2-*mpaR-GFP and* pPL2-*mpaR-H54A-GFP)* were delivered by conjugation into Δ*mon/*$\Delta\phi$ *L. monocytogenes* bacteria. The strains were grown at 30°C in BHI supplemented with 100 ng/ml of anhydrotetracycline. Total protein content was extracted and assayed using a modified Lowry assay, and samples with equal amounts of total proteins (20 µgr) were separated on 12.5% SDS-polyacrylamide gels and transferred to nitrocellulose membranes. Tagged proteins were probed with rabbit anti-GFP antibody, followed by HRP-conjugated goat anti-mouse IgG. The developed Western blot images were analyzed with ImageJ to quantify bands intensity. The intensities of the bands corresponding the mutated protein were normalized to that of the native protein (mut/native). The data represents the mean and stranded deviation of 3 independent biological repeats. Source data are provided as a Source Data file.

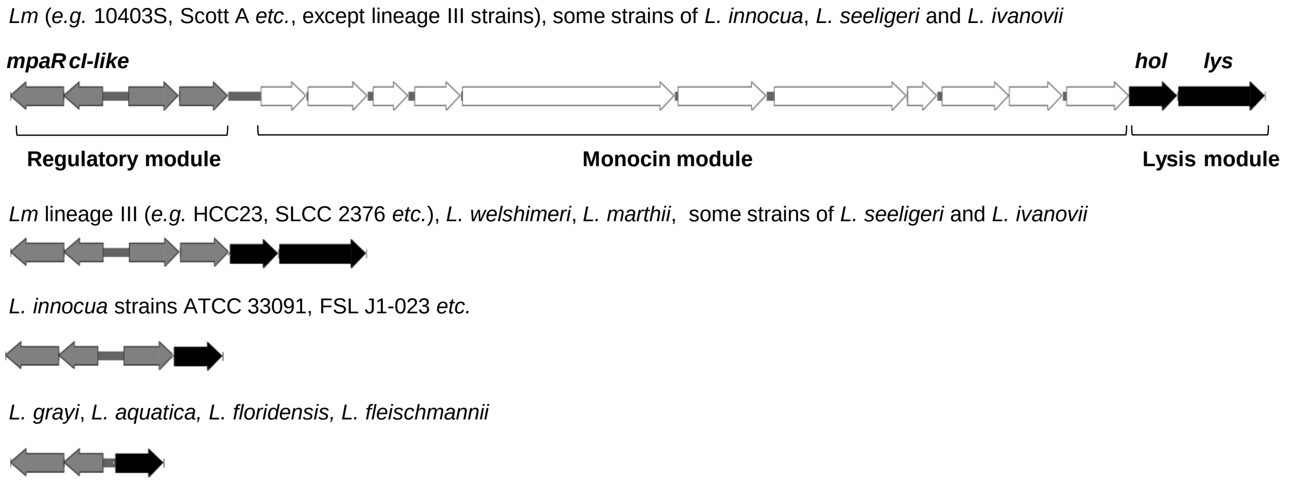

Supplementary Figure 5. Schematic representation of the monocin locus in different *Listeria* strains. While some strains harbor a cluster of 17 genes (the longest version), others contain 6, 4, and 3 genes, all including the *mpaR* gene.

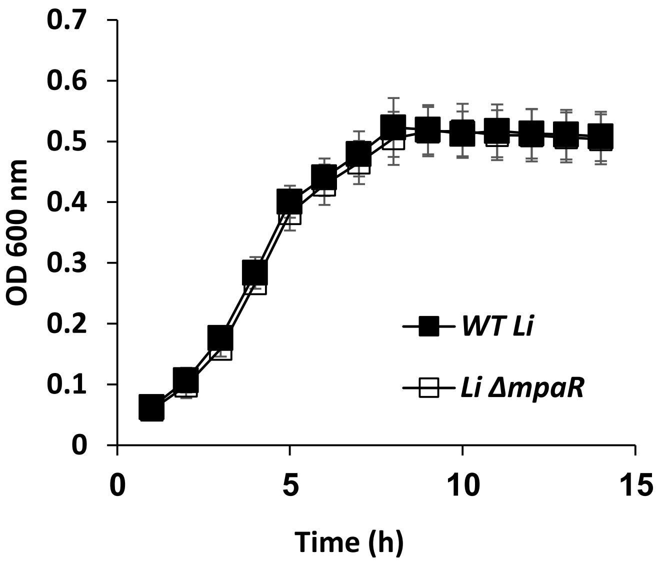

Supplementary Figure 6. Growth analysis of WT *Li* and *Li*-Δ*mpaR* in the absence of MC. Growth analysis of WT *L. innocua* (WT *Li*) and its isogenic *mpaR* mutant (*Li*-Δ*mpaR*) without MC. The data shows the mean and the standard deviation of three independent biological repeats. Complementary to **Fig 7C**. Source data are provided as a Source Data file.

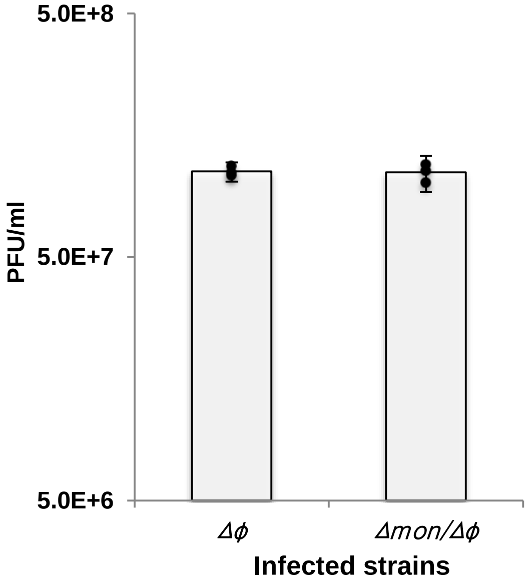

Supplementary Figure 7. ϕ10403S lytic infection is independent of the monocin. A plaque forming assay using ϕ10403S phage infecting *Δϕ* and *Δmon/Δϕ* bacteria. Virions were obtained from WT *Lm* bacteria treated with MC, and were used to infect indicated strains on soft agar plates, where plaques were visible to count (see materials and methods). Error bars represent the standard deviation of 3 independent experiments. Source data are provided as a Source Data file.

Listeria strains	Access. No	MpaR-like DUF995	CI-like	AAH cleavage site in CI-like repressors
Type-I (39 strains)				
01-1280 (1/2a)	CP006940.1	+	+	+
01-1468 (1/2a)	CP007527.1	+	+	+
03-5473	CP008773.1	+	+	+
08-5578 (1/2a)	CP001602.2	+	+	+
08-5923 (1/2a)	CP001604.1	+	+	+
10-0814	CP008836.1	+	+	+
10-0815 (1/2a)	CP006860.1	+	+	+
10-5025 (1/2c)	CP007194.1	+	+	+
10-5026 (1/2c)	CP007195.1	+	+	+
10-5027 (3c)	CP007196.1	+	+	+
99-6370 (1/2a)	CP007021.1	+	+	+
CFSAN042079	CP019170.1	+	+	+
FORC_057	CP021174.1	+	+	+
HPB913	CP018685.1	+	+	+
HPB2088	CP019164.1	+	+	+
HPB5415	CP019165.1	+	+	+
HPB5622	CP019167.1	+	+	+
J0161 (1/2a)	CP002001.1	+	+	+
J2-031	CP006593.1	+	+	+
Lm60 (1/2a)	CP009258.1	+	+	+
NH1 (1/2c)	CP021325.1	+	+	+
10-092876-0055 LM4 (1/2a)	CP019617.1	+	+	+
10-092876-0731 LM5 (1/2a)	CP019618.1	+	+	+
J1776 (4b-like)	CP006598.1	+	+	+
J1816 (4b)	CP006047.2	+	+	+
J1817 (4b-like)	CP006599.1	+	+	+
J1926 (4b-like)	CP006600.1	+	+	+
Lm 3136	CP013723.1	+	+	+
10-0810 (1/2b)	CP007168.1	+	+	+
10-0811 (1/2b)	CP007169.1	+	+	+
10-0819	CP008768.1	+	+	+
CFSAN008100	CP011398.2	+	+	+
FDA00006905	CP023052.1	+	+	+
FDA00006907	CP022020.1	+	+	+
FDA00011238	CP023050.1	+	+	+
FW040025 (1/2a)	CP011345.1	+	+	+
Lm N1546	CP013724.1	+	+	+
MOD1_LS152	CP020830.1	+	+	+
R479a (1/2c-like)	HG813247.1	+	+	+
Type-II (22 strains)				
10403S (1/2a)	CP002002.1	-	+	+
EGD-e (1/2a)	NC_003210.1	-	+	+
10-0812 (1/2a)	CP007170.1	-	+	+
10-0813 (1/2a)	CP007171.1	-	+	+
10-092876-0168	CP019615.1	-	+	+
10-092876-0769 LM12	CP019625.1	-	+	+
AT3E (1/2c)	CP023752.1	-	+	+
CFSAN029793	CP016213.1	-	+	+
CFSAN023459	CP014252.1	-	+	+
CFSAN028538	CP020827.1	-	+	+
FSL R2-56n1 (1/2c)	CP002003.1	-	+	+
L2676 (1/2a)	CP007685.1	-	+	+
MF4624	CP025259.1	-	+	+
MF4626	CP025082.1	-	+	+
MF4697	CP025438.1	-	+	+
MF6172	CP025440.1	-	+	+
PIR00540	CP025568.1	-	+	+
PIR00544	CP025565.1	-	+	+
R2-502 (1/2b-like)	CP006594.1	-	+	+
SLCC 2372 (1/2c)	NC_018588.1	-	+	+
SLCC 2479 (3c)	NC_018589.1	-	+	+
L. innocua Clip11262 (6a)	NC_003212.1	-	+	+
Type-III (7 strains)				
AL4E (1/2c)	CP023754.1	+	+	-
ATCC 51779 (1/2c)	CP025567.1	+	+	-
C1-387 (1/2c)	CP006591.1	+	+	-
CFSAN054109	CP028183.1	+	+	-
F4244	CP015508.1	+	+	-
ICDC-LM188	CP015593.1	+	+	-
L1846	CP007688.1	+	+	-

Supplementary Table 1. Distribution of *mpaR-like* (DUF955 family) and repressor (*cI-like*) genes in *comK* prophages*, Listeria* species. Only complete genomes of *Listeria* strains were used for the analysis (Mar 24-25, 2019). *L. monocytogenes* strains are shown without the name of the species. A serotype name shown in brackets. Two groups of prophages were identified: Type I and Type III- encoding MpaR and CI-like proteins, Type II –encoding for CI-like protein without an MpaR-like homolog. An AAH cleavage site is present in Type I and II and missing in Type III. Species names are in italics when appropriate.
